# Supplementary material for: Computational single-neuron mechanisms of visual object coding in the human temporal lobe
Source: Nat Commun. 2026 Feb 1;17:2234. doi: 10.1038/s41467-026-68954-8 (PMC12963616; doi:10.1038/s41467-026-68954-8)
Supplement: Supplementary file 1 — Supplementary Information [file 41467_2026_68954_MOESM1_ESM.pdf]

Supplementary Information

Supplementary Results

Category-selective neural response

We identified channels that showed significant differential responses to the 50 object categories (see representative channels in **Supplementary Fig. 1a**) using a one-way ANOVA and an additional response threshold criterion (see **Methods** for details). We observed a significant number of category-selective channels (binomial $P < 0.05$) in all subregions of the VTC and MTL, except in the left PH (**Supplementary Fig. 1b**). Interestingly, the left ITG (25.49%) had a significantly higher proportion of category-selective channels compared to the right ITG (13.01%; χ^2 test: $P = 0.01$), suggesting lateralized processing of object categories in the ITG. We further confirmed the results using a category selectivity index (d' between the most-preferred and least-preferred categories; **Supplementary Fig. 1c**) and a depth of selectivity (DOS) index (**Supplementary Fig. 1d**). In addition, channels selective for multiple categories were defined as MC (multiple categories) channels, while those selective for a single category as SC (single category) channels. When ranking responses from the most-preferred to the least-preferred category, as expected, SC channels showed a sharp decrease in response after the most-preferred category, whereas MC channels exhibited a more gradual decline from the most-preferred to the least-preferred category (**Supplementary Fig. 1e**). This effect was further evidenced by a larger decrease in neural response between the most-preferred and second most-preferred stimuli in SC channels compared to MC channels (**Supplementary Fig. 1f**; two-tailed two-sample t-test: $t(346) = 8.24$, $P = 3.50\times10^{-15}$, $d = 0.93$, 95% CI = [0.022, 0.035]). Furthermore, we measured the onset latency for each category-selective channel (**Supplementary Fig. 1g**) and found that the FG exhibited the earliest category-selective response (219.5 ± 187.4 ms), followed by the amygdala (246 ± 312.4 ms), AH (300.5 ± 186.3 ms), and PH (473.5 ± 175.8 ms).

Supplementary Discussion

Advantages of our study and possible caveats

Previous research on the computational neural mechanisms of visual object coding has primarily focused on individual brain areas, particularly in the higher visual cortex of non-human primates [1-3]. Human iEEG studies generally include only a few object categories to examine the neural mechanisms for object recognition [4-6]. However, few studies have investigated the neural pathway for object processing using diverse and naturalistic stimuli, especially at the single-neuron and neural circuit levels in the human brain. In this study, we employed an "object space" approach [7-12] to comprehensively investigate the spatial organization and temporal dynamics of object coding. Notably, we used DNNs to build neural models. DNNs can extract visual features from naturalistic images of objects, enabling us to study the neural coding of complex, real-world stimuli. Both neuroimaging [13] and electrophysiology [14] studies have shown that DNN features can be represented in the human brain, and monkey studies have shown that images synthesized by DNNs can control neural population activity [3,15].

In this study, we primarily recorded from the anterior parts of the FG (268 out of 278 channels) and ITG (390 out of 414 channels) based on the electrode coverage of our patients. The anterior temporal cortex plays a significant role in visual object processing [16], particularly in encoding the abstract conceptual properties of objects [17,18]. However, due to signal distortion, it has been under-explored in neuroimaging studies [19,20]. Our unique opportunity to directly record neural activity in this area sheds new light on its role in visual object processing. However, our recordings did not include the posterior FG and ITG, which have been more classically studied in neuroimaging studies [21,22]. This limited our ability to examine object coding along the visual hierarchy within the FG.

Our RSA revealed significant correlations between axis-coding channels in the VTC and category-coding channels in the MTL (**Fig. 3h**). The relatively low correlation values likely reflect the related yet distinct nature of neural representations in the VTC and MTL: axis coding emphasizes visual features, whereas category coding integrates higher-level semantic information. The transformation between these coding schemes is complex and nonlinear, introducing variability that reduces direct similarity. Additionally, methodological factors—such as noise, inter-subject variability, and spatial resolution—may contribute to the lower correlation values. Despite this, the statistically significant correlations indicate a meaningful relationship, supporting the role of axis coding as an intermediate step in the transition from visual to categorical representations. Interestingly, we observed that the axis-coding RDM in the FG exhibited a more prominent block structure than some of the category-coding RDMs in the MTL (**Supplementary Fig. 5**). Although this may seem counterintuitive, it is consistent with the

idea that feature-based representations derived from naturalistic visual input can preserve underlying semantic regularities. In contrast, category-coding signals—particularly in MTL regions—may operate in a more selective or sparse manner, prioritizing categorical boundaries over continuous similarity structure. This distinction suggests that axis coding in perceptual regions such as the FG may support rich, graded encoding of visual similarity, while category coding in the MTL may support more abstract, discrete representations that facilitate memory and decision making. Together, these complementary coding schemes likely contribute to the transformation from perceptual to conceptual representations along the ventral visual pathway.

We observed comparable region coding across MTL subregions (PH, AH, and amygdala). While these regions are associated with distinct cognitive and affective functions, extensive literature has shown that they share commonalities in the visual processing of faces and objects [23-29]. Our previous studies directly comparing face [30] and object [29] coding in the amygdala and hippocampus also found qualitatively similar results between these regions, making our present findings highly consistent with the literature. This similarity may stem from a common input, such as the FG and/or ITG, as supported by the similar functional connectivity of the amygdala and hippocampus with the VTC (**Fig. 6**; **Fig. 7**). While there were differences in information flow between VTC and MTL subregions (**Fig. 3h**), as well as dissociations in frequency-specific PLV patterns across MTL subregions (**Fig. 6a, b**), it is important to note that the increase in phase synchronization for axis-coding channels was not confined to any single MTL subregion (**Fig. 6b**). Instead, this widespread PLV enhancement may reflect a more global, network-level coordination pattern driven by axis-based coding in the VTC. These findings suggest that axis-based coding in the VTC may serve as a unifying mechanism for broadcasting perceptual information across the MTL, facilitating integrated memory representations. Rather than operating in isolation, MTL subregions may be dynamically coordinated by shared input from feature-coding regions, allowing for distributed yet coherent processing of visual information.

When selecting the DNN layer for visual feature extraction (**Supplementary Fig. 2a**), we found that layer pool5 consistently provided the best overall fit to neural data across brain areas. However, intermediate layers (e.g., res3b3) also achieved relatively high R^2 values in the test dataset, particularly in certain brain areas. This pattern suggests that features from mid-level layers may, in some cases, better capture the representational structure of neural responses. Moreover, the larger discrepancy

between the full and test datasets observed for higher layers indicates that these deeper representations, though highly predictive during training, may exhibit reduced generalizability to novel data.

Axis coding

While our axis-coding framework primarily captures continuous visual feature dimensions, we note that in some cases, these axes may align more closely with categorical distinctions. For example, the stimulus distribution shown in **Fig. 7g** suggests that certain categories cluster along the preferred axis of the ITG channel, raising the possibility that axis coding may, in some instances, reflect categorically meaningful groupings. This could result from shared visual features within categories (e.g., similar shapes or parts) or from the progressive transformation of perceptual representations into more abstract, category-like formats along the VTC hierarchy. On the other hand, it is worth noting that there was parametric variation along given axes within a single category, suggesting that axis coding preserves fine-grained visual differences even among exemplars of the same category. For instance, stimuli from the same object class may appear distributed along the axis in a gradient-like fashion, reflecting systematic changes in shape, size, or configuration (**Fig. 3a, b**). This supports the notion that axis coding does not merely represent categorical labels but captures a continuous feature space that underlies both within- and between-category structure. Together, these observations highlight the dual nature of axis coding in the VTC: while it may incidentally align with categorical groupings due to feature regularities, it fundamentally reflects graded visual dimensions that enable nuanced object discrimination. Future work could apply feature decomposition analysis to further elucidate the representational content of these axes and to examine the diversity of this content across VTC subregions.

Functional connectivity

We observed region-based coding at the microscopic single-neuron level, but not at the mesoscopic iEEG level. The absence of region coding in MTL iEEG channels may be attributed to two factors. First, region coding might occur at the single-neuron level, with neighboring neurons encoding different regions that are not adjacent in the feature space. Consequently, an iEEG channel—pooling activity from thousands of neurons—could mask fine-scale region-based tuning. Second, the current region-coding

algorithm may be more sensitive to sparse single-neuron responses with high stimulus-dependent variance, whereas iEEG activity typically exhibits less variation across stimuli. These methodological limitations may also apply to VTC channels, potentially preventing the detection of certain types of coding that occur only at fine spatial scales. Therefore, direct comparisons between VTC and MTL based solely on iEEG data should be made with caution, as differences in observed coding schemes may partly reflect disparities in detectability rather than true functional divergence. Future studies employing single-neuron recordings in both regions will be essential to resolve these issues and to validate the representational formats inferred from mesoscopic signals.

In this study, we observed phase-locking at lower frequencies, while Granger causality was present at higher frequencies. This difference in frequency ranges may arise from the distinct neural mechanisms captured by these two methods. Specifically, phase-locking at lower frequencies (e.g., delta, theta, and alpha bands) between the VTC and MTL suggests that these regions engage in synchronized oscillatory activity, potentially supporting integrative processes such as memory consolidation or long-range communication. In contrast, our Granger causality analysis revealed directed connectivity at higher frequencies (e.g., beta and gamma bands), which may be associated with fast-paced information transfer and local computations. Therefore, the discrepancy between phase-locking and Granger causality frequencies likely reflects complementary aspects of VTC-MTL interactions: lower-frequency synchrony may underlie broad-scale coordination, while higher-frequency connectivity supports efficient and rapid information transfer. Future studies are needed to further differentiate these neural processes.

The FG and ITG exhibited stronger feedforward Granger causality (GC) to the AH in the low-frequency range, suggesting that low-frequency oscillations may serve as a mechanism for transmitting visual information from the VTC to the MTL during object perception. Interestingly, this effect was more pronounced in the spectral GC analysis (**Fig. 6d**) than in the time-resolved GC analysis (**Fig. 6e**). This discrepancy may reflect differences in the sensitivity of these methods—spectral GC is better suited for detecting sustained frequency-specific interactions, whereas time-resolved GC emphasizes transient, temporally localized effects. Additionally, the feedforward influence may be more specific to the AH, with weaker effects in other MTL subregions such as the PH or amygdala. These findings highlight the importance of combining multiple analytical approaches and considering subregional specificity within the MTL when examining directed functional connectivity in the human brain.

Shared computational mechanisms for face and object coding in the VTC

While the present study demonstrated computational principles for object stimuli—specifically, how dense, feature-based representations in the VTC are transformed into sparse, category-based representations in the MTL—faces share similar computational principles [31]. This aligns with previous macaque studies demonstrating shared axis coding mechanisms for both faces [32] and objects [10]. Using data from a subset of 11 patients (14 sessions) in which both object and face stimuli were recorded (in separate sessions), our analyses revealed that axis coding in the VTC is not specific to face-selective channels (even within the FG), as face-selective sites were no more likely to exhibit axis coding than the broader channel population. Notably, a substantial proportion of channels showed axis coding for both faces and objects, indicating that axis coding generalizes across stimulus categories. This pattern held both across the VTC and specifically within the FG, where many channels that encoded objects along axes also did so for faces. Therefore, although there are nuanced differences between object and face coding—for example, the ITG was not involved in axis coding for faces [31] but was for objects; some channels exhibited face selectivity; and some channels exhibited axis coding exclusively for faces—similar computational mechanisms appear to underlie the encoding of both object and face stimuli. Furthermore, we found that a large majority of category-selective channels also exhibited axis coding, supporting the idea that axis coding serves as a general representational format in the higher visual cortex.

Response latency

The time window (0.1–0.6 s relative to stimulus onset) used for the iEEG analysis was selected based on the observed temporal profile of the response (**Fig. 2e**), which is consistent with the early PLV synchronization observed in **Fig. 6b**. To assess the robustness of our findings, we repeated the main analyses using an extended time window (0–1 s relative to stimulus onset) and found that the results remained consistent. In contrast, the MTL spike–VTC LFP coupling (**Fig. 7d**) emerged at a later latency (~400 ms), in line with the spiking profiles of MTL neurons reported in our previous studies [29,30,33-35]. Therefore, we employed a longer time window (0.25–1.25 s relative to stimulus onset) for the single-

neuron analyses in the current study. Collectively, the temporal windows used for both iEEG and single-neuron analyses were selected to align with the distinct temporal dynamics of their respective signals, ensuring that the analyses were appropriately tailored to the underlying neural response profiles.

While the AH showed significant axis coding at the mesoscopic iEEG level, its late-onset latency (**Fig. 3f**) may indicate a closer alignment with region coding, as region coding inherently involves encoding localized receptive fields in the VTC feature space and may share similar temporal characteristics with late-onset axis coding. Computationally, we also demonstrated that an elevated response at the border of the feature space can drive both axis coding and region coding [30]. However, it is important to note that we did not observe a significant number of iEEG channels exhibiting region coding in the AH, suggesting that axis coding may better account for the iEEG responses in this region. Future studies are therefore needed to directly compare the onset latencies of axis and region coding in the AH and more broadly along the VTC–MTL pathway.

The late onset of MTL spike–VTC field coupling (**Fig. 7d**) likely reflects a later stage of cognitive processing associated with higher-level integration rather than initial visual encoding. Notably, the timing of this coupling aligns closely with the delayed axis coding observed in the AH (**Fig. 3f**), suggesting a potential link between these two neural phenomena. This temporal correspondence raises the possibility that the spike–LFP coupling observed in **Fig. 7d** is functionally related to the emergence of high-level representational coding in the AH. Taken together, these findings support the existence of a multi-stage processing hierarchy, wherein early neural responses (0.1–0.4 s post-stimulus) are primarily driven by bottom-up visual feature encoding, while later responses (from ~400 ms onward) reflect top-down, associative processes involving mnemonic integration.

Lastly, the differences in response latency may be related to differences in oscillatory frequency. In contrast to the early onset (<100 ms) of VTC–MTL interactions observed at lower frequencies in mesoscopic iEEG signals (**Fig. 6b**), neurons in the MTL fired in coordination with VTC axis-coding iEEG channels at a later latency (~400 ms) and in higher-frequency ranges (**Fig. 7d**). The distinct temporal and spectral profiles revealed by signals from different spatial scales may reflect different stages of object perception. Further studies are needed to investigate how inter-areal synchronization varies across spatial and temporal scales to gain a comprehensive understanding of functional connectivity during object perception.

Alternative interpretations of our computational framework

An alternative interpretation of the VTC-to-MTL transformation is that the observed representational changes reflect a general hierarchical integration process, rather than a dedicated transformation. Similar to how receptive fields in early visual areas converge to form higher-order visual representations in regions such as V4 and IT, the emergence of region-based coding in the MTL may arise from the cumulative integration of visual features represented in the VTC. In this view, the clustering of neural responses in MTL—sometimes aligning with object categories—could result from overlapping visual feature selectivity, rather than an explicit coding of semantic content. This interpretation is consistent with known principles of hierarchical organization in sensory systems and suggests that apparent semantic structure may emerge naturally from the integration of complex visual information, even in the absence of explicit category representations. Our findings that region-based tuning in the MTL transcends strict category boundaries further support this view. Future work will be critical to determine whether the transformation from perceptual to conceptual representations in the MTL reflects purely integrative processes or additional mechanisms that encode abstract conceptual meaning.

Another important question concerns whether region-based coding in feature space, as observed in the MTL, might originate earlier in the ventral visual stream—specifically in regions such as the FG or ITG—and simply be inherited by the MTL. While our model (**Fig. 1**) posits that such region-based coding emerges in the MTL, the current study does not include single-neuron recordings from the FG or ITG and therefore cannot directly rule out this possibility. However, we addressed this issue in a separate experiment using the same paradigm (restricted to face stimuli), in which we recorded multi-unit activity (MUA) from the inferotemporal cortex (ITC) of a macaque monkey [30,31]. The ITC is a well-established homolog of the human VTC, encompassing both the FG and ITG. Applying the same computational framework to assess the presence of axis and region coding in this dataset, we found robust and prevalent axis coding across all 53 MUA channels (100%; strength of axis coding = 0.48 ± 0.13 [mean ± SD]), while region coding was observed in a significantly smaller fraction of neurons (30.2%; χ^2 test: $P = 4.73\times10^{-14}$), and this effect was likely secondary to the axis structure [30,31]. These findings suggest that axis-based representations dominate in the ITC/VTC, while region-based coding emerges more prominently in the MTL. Therefore, it is unlikely that region coding observed in the MTL is simply

inherited from the VTC. Instead, our results support a transformation in representational format between the VTC and MTL.

ID	Sex	Age	Race	Epilepsy diagnosis	Number of channels									
					VTC				MTL					
					FG		ITG		PH		AH		Amygdala	
					L	R	L	R	L	R	L	R	L	R
BJH024	F	38	Caucasian	Left temporal	6	7	9	8	2	3	3	0	5	2
BJH025	F	45	Caucasian	Right mesial temporal	0	12	1	17	0	5	1	1	5	9
BJH026	M	40	Caucasian	Left mesial temporal	14	0	13	0	1	0	0	0	3	5
					14	0	13	0	1	0	0	0	3	5
BJH027	M	37	Caucasian	Right temporal	9	7	23	14	2	3	4	6	4	5
					9	7	23	14	1	3	4	6	4	5
BJH028	F	36	Caucasian	Left frontal SMA, cingulate regions	0	0	0	0	0	0	0	0	3	0
BJH029	F	34	Caucasian	Left amygdala and hippocampus	15	0	17	1	5	0	5	5	5	5
					15	0	18	1	5	0	5	5	5	5
BJH030	F	22	Caucasian	Right amygdala and hippocampus	10	7	19	16	8	3	8	0	5	3
					10	7	19	16	8	7	4	0	5	2
BJH032	M	27	Caucasian/ American Indian	Bilateral amygdala and hippocampus	12	8	8	9	6	3	1	2	5	4
					12	8	8	9	6	3	1	2	5	3
BJH033	F	24	Caucasian/ American Indian	Right hippocampus	1	7	1	18	0	4	2	1	3	6
					1	10	1	20	0	5	2	2	3	6
BJH037	M	46	Caucasian	Bilateral amygdala and hippocampus	7	6	15	11	2	0	3	2	7	5
BJH050	F	28	Caucasian	Right temporal	0	15	1	14	0	4	5	3	0	7
BJH051	F	41	Caucasian	Left amygdala and hippocampus	8	1	11	0	2	0	4	1	6	3
BJH052	M	24	Caucasian	Bilateral hippocampus	8	8	19	3	4	0	6	2	6	3
					8	8	19	3	4	0	6	2	6	3
BJH053	F	20	Caucasian	Right middle frontal	0	1	0	2	0	0	0	1	0	3
	Sum				159	119	238	176	57	43	64	41	88	89

Supplementary Table 1. Patients and sessions for iEEG recordings of the ImageNet stimuli. VTC: ventral temporal cortex. MTL: medial temporal lobe. FG: fusiform gyrus. ITG: inferior temporal gyrus. AH: anterior hippocampus. PH: posterior hippocampus. L: left. R: right.

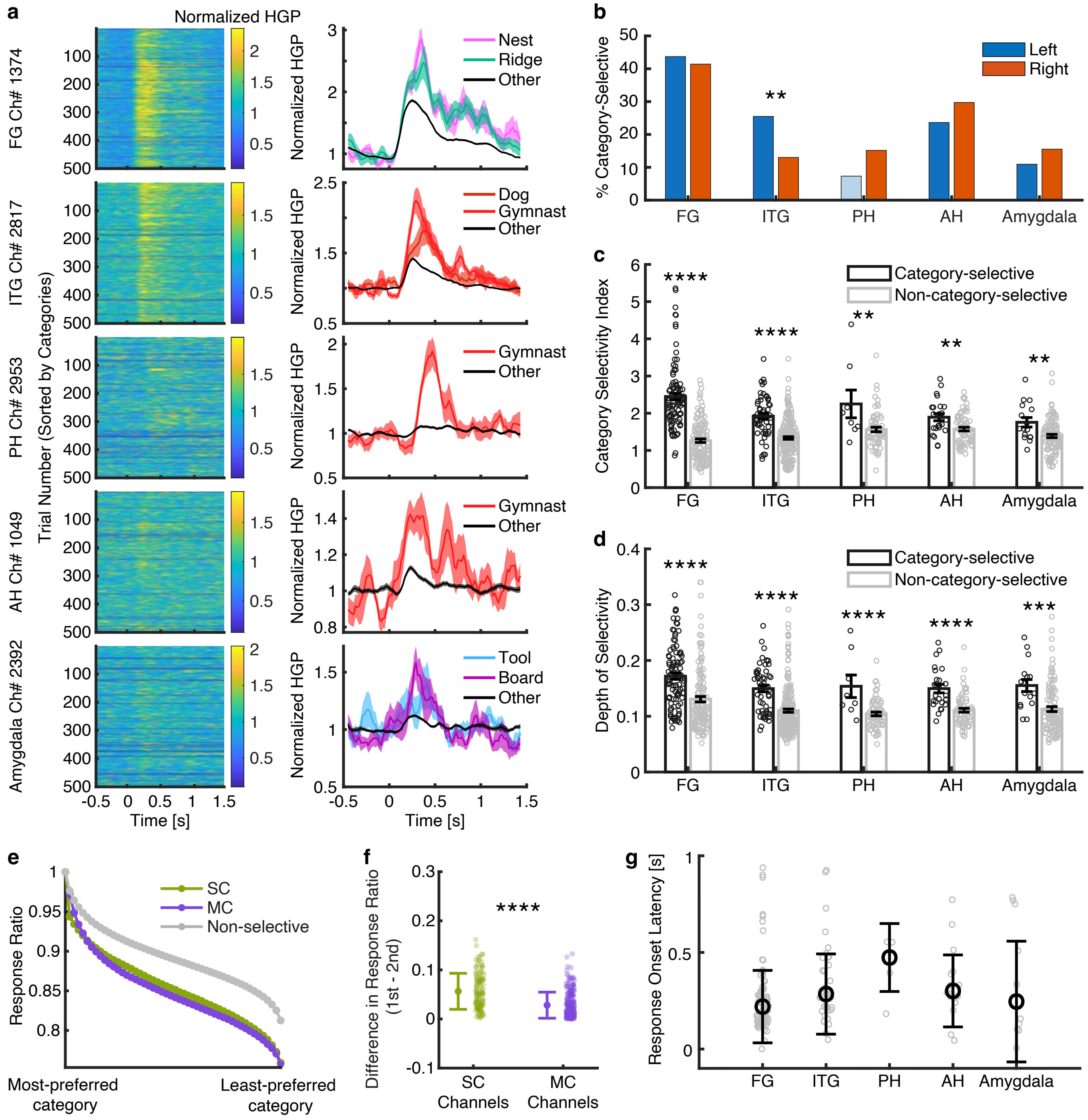

Supplementary Fig. 1. Category-selective coding. **(a)** Representative category-selective channels. (left) Single-trial response profiles, with trials sorted by object categories. Each row is a trial. Color coding indicates baseline-normalized high-gamma power (HGP). Time 0 denotes trial onset. (right) Mean response across trials. The object categories selectively encoded by the channel are shown separately using different colors. Shaded area denotes ±SEM across trials. **(b)** Percentage of category-selective channels in each ROI, separately for the left and right hemispheres. χ^2-test was applied to compare the

percentage between the left and right ROIs. **: $P < 0.01$. **(c)** Category selectivity index. To assess each channel's selectivity to different object categories, we defined a category selectivity index as the d' between the most-preferred and least-preferred object categories. A similar index was used in previous studies to assess the level of selectivity to different faces [14,30]. It is worth noting that the category selectivity index was not used to select category-selective channels or estimate the number of channels that were category-selective. Instead, the category selectivity index was used to quantify the degree of category selectivity for the category-selective and non-category-selective channels that had already been selected. **(d)** Depth of selectivity. Each circle represents an individual channel. Error bars denote ±SEM across channels. Asterisks indicate a significant difference between category-selective and non-category-selective channels using a two-tailed two-sample *t*-test. **: $P < 0.01$, ***: $P < 0.001$, and ****: $P < 0.0001$. **(e)** Ordered average responses from the most- to the least-preferred category. Non-category-selective channels are shown for comparison purposes. Responses were normalized by the response to the most-preferred category. Shaded areas denote ±SEM across channels. **(f)** Difference in response ratio between the most-preferred and second most-preferred categories. Each dot represents an individual channel. Error bars denote ±SEM across channels. Asterisks indicate a significant difference between single-category (SC) and multiple-category (MC) channels. ****: $P < 0.0001$. **(g)** Response onset latency of category-selective channels for each ROI. Each gray circle represents an individual channel. The black circle represents the median, and error bars denote ±SD across channels. Source data are provided as a Source Data file.

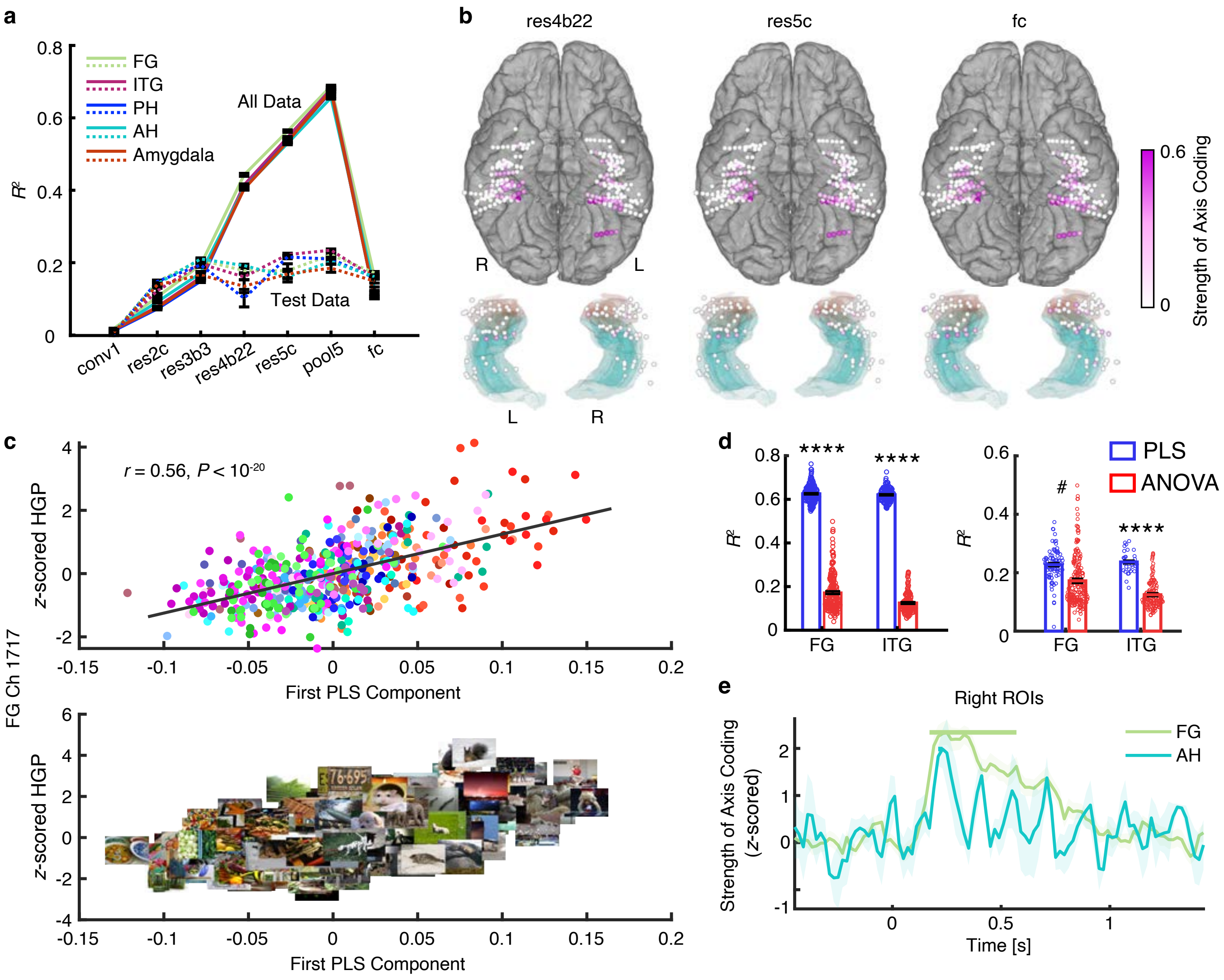

Supplementary Fig. 2. Additional results for axis coding. **(a)** Goodness-of-fit (R^2) of the partial least squares (PLS) regression with deep neural network (DNN) features for each DNN layer. The layer with the highest R^2 was selected for further analysis. Error bars denote ±SEM across channels. R^2 was calculated using both the full dataset (solid line) and a held-out test dataset (dotted line; see **Methods**). Both datasets indicated that layer pool5 provided the best fit to the neural responses. **(b)** Distribution of axis-coding channels selected using DNN features from layers with above-chance selection. Color coding shows the strength of axis coding. It is worth noting that panel **(a)** shows R^2 values for all visually responsive channels, calculated using either the full dataset or a held-out test dataset, whereas panel **(b)** depicts the strength of axis coding, quantified as the correlation between observed and predicted responses in the held-out test dataset. The predicted responses in **(b)** were generated using models trained only on the training dataset (see Methods for details). The apparent discrepancy—where layers such as res4b22, res5c, and fc differ in R^2 but show little difference in Pearson's r—likely reflects

fundamental differences between these metrics. R^2 is sensitive to both bias and scaling, capturing how well the model explains variance in the dataset, whereas the correlation coefficient emphasizes generalization performance and is less sensitive to absolute scaling differences. In summary, R^2 values are most informative for comparing relative model fits across feature layers, while correlation coefficients (i.e., the strength of axis coding) reflect the models' ability to generalize to unseen data. **(c)** An example channel in the fusiform gyrus (FG) representing the axis of animacy. Images were obtained from Deng J, Dong W, Socher R, Li L-J, Li K, Fei-Fei L (2009). IEEE Conference on Computer Vision and Pattern Recognition 2009: 248–55. IEEE. **(d)** R^2 for the axis-coding PLS model and category-coding ANOVA model. Each circle represents a visually responsive channel, and error bars denote ±SEM across channels. Asterisks indicate a significant difference between models using a two-tailed paired *t*-test. #: $P < 0.1$, and ****: $P < 0.0001$. (left) R^2 calculated using the full dataset. (right) R^2 calculated using a held-out test dataset. It is important to note that ANOVA does not involve training/testing procedures or cross-validation; its R^2 was computed directly from the full dataset. **(e)** Temporal profile of axis coding among the significant ROIs from the right hemisphere. Legend conventions as in **Fig. 3**. Source data are provided as a Source Data file.

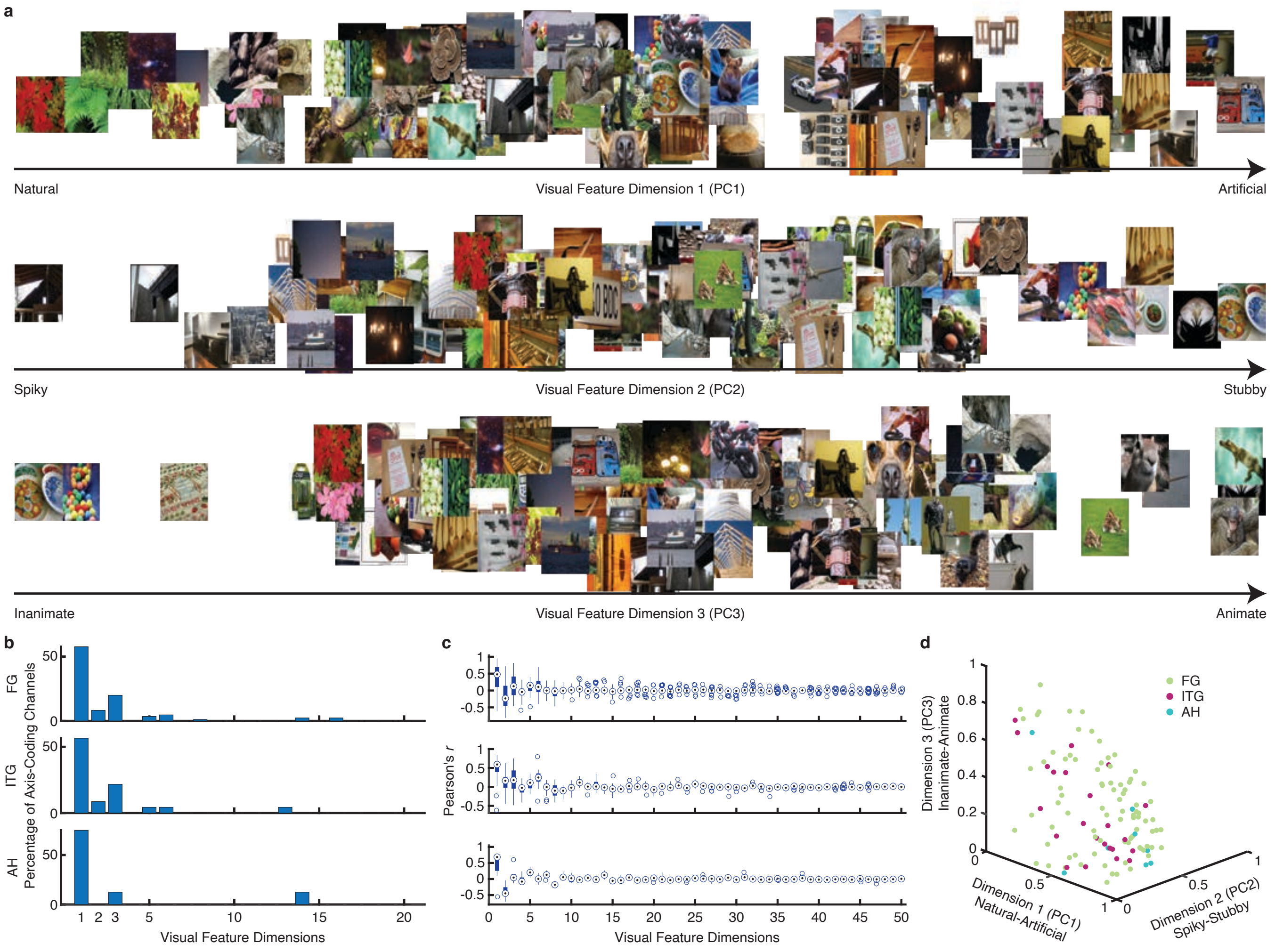

Supplementary Fig. 3. Summary of the encoded feature axes for the ImageNet dataset. **(a)** Visualization of the first three visual feature dimensions (principal components [PCs] of the object visual features). These PCs represent the well-known natural–artificial, spiky–stubby, and inanimate–animate dimensions. Images were obtained from Deng J, Dong W, Socher R, Li L-J, Li K, Fei-Fei L (2009). IEEE Conference on Computer Vision and Pattern Recognition 2009: 248–55. IEEE. **(b)** Percentage of axis-coding channels aligned with each visual feature dimension. For each axis-coding channel, we first identified its neural tuning axis from the PLS regression. The best-aligned visual feature dimension was then determined by computing the correlation between the neural axis of a given channel and each of the visual feature dimensions. **(c)** Correlation coefficients between the neural tuning axes and visual feature dimensions. In each box plot, the central mark represents the median, box edges indicate the 25th and 75th percentiles, whiskers extend to non-outlier extremes, and circles denote outliers. **(d)** Distribution of axis-coding channels in the visual feature space, constructed using three principal visual feature dimensions. Colors represent different axis-coding brain areas. FG: fusiform

gyrus. ITG: inferior temporal gyrus. AH: anterior hippocampus. Source data are provided as a Source Data file.

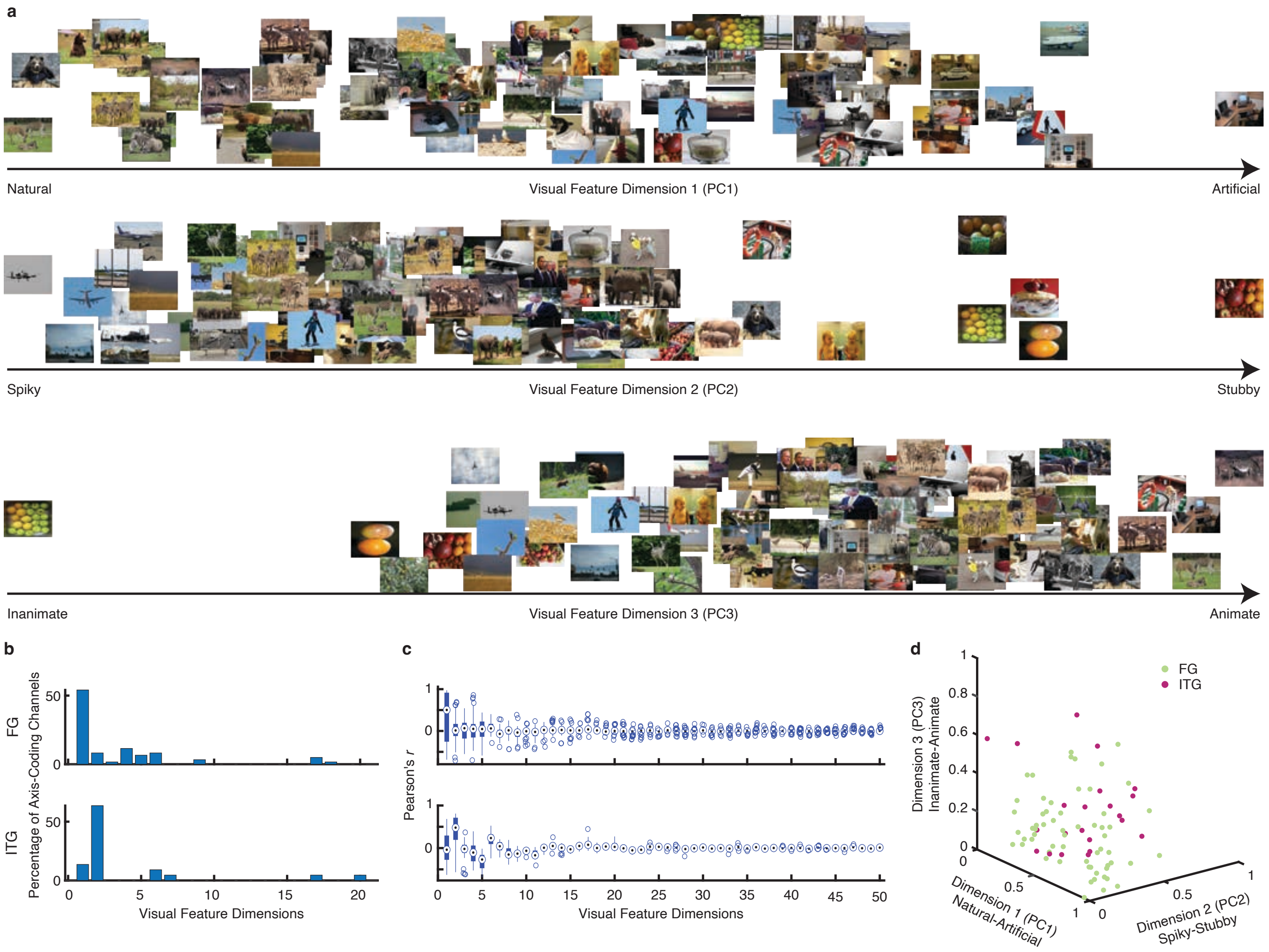

Supplementary Fig. 4. Summary of the encoded feature axes for the Microsoft COCO dataset. **(a)** Visualization of the first three visual feature dimensions (principal components [PCs] of the object visual features). Images were obtained from Lin T-Y, Maire M, Belongie S, Hays J, Perona P, et al. (2014). Microsoft COCO: Common Objects in Context, pp. 740–55. Cham: Springer International Publishing. **(b)** Percentage of axis-coding channels aligned with each visual feature dimension. **(c)** Correlation coefficients between the neural tuning axes and visual feature dimensions. **(d)** Distribution of axis-coding channels in the visual feature space, constructed using three principal visual feature dimensions. Legend conventions as in **Supplementary Fig. 3**. Source data are provided as a Source Data file.

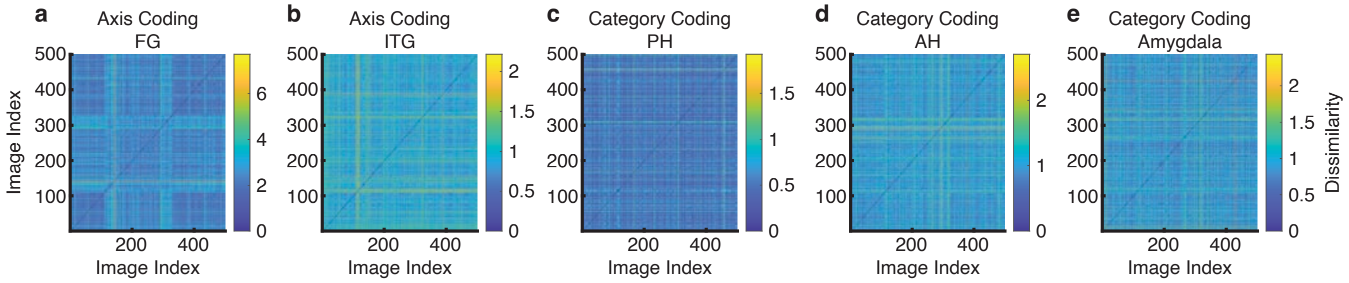

Supplementary Fig. 5. Representational dissimilarity matrix for each ROI. **(a, b)** Axis-coding channels in the ventral temporal cortex (VTC). **(c-e)** Category-coding channels in the medial temporal lobe (MTL). Source data are provided as a Source Data file.

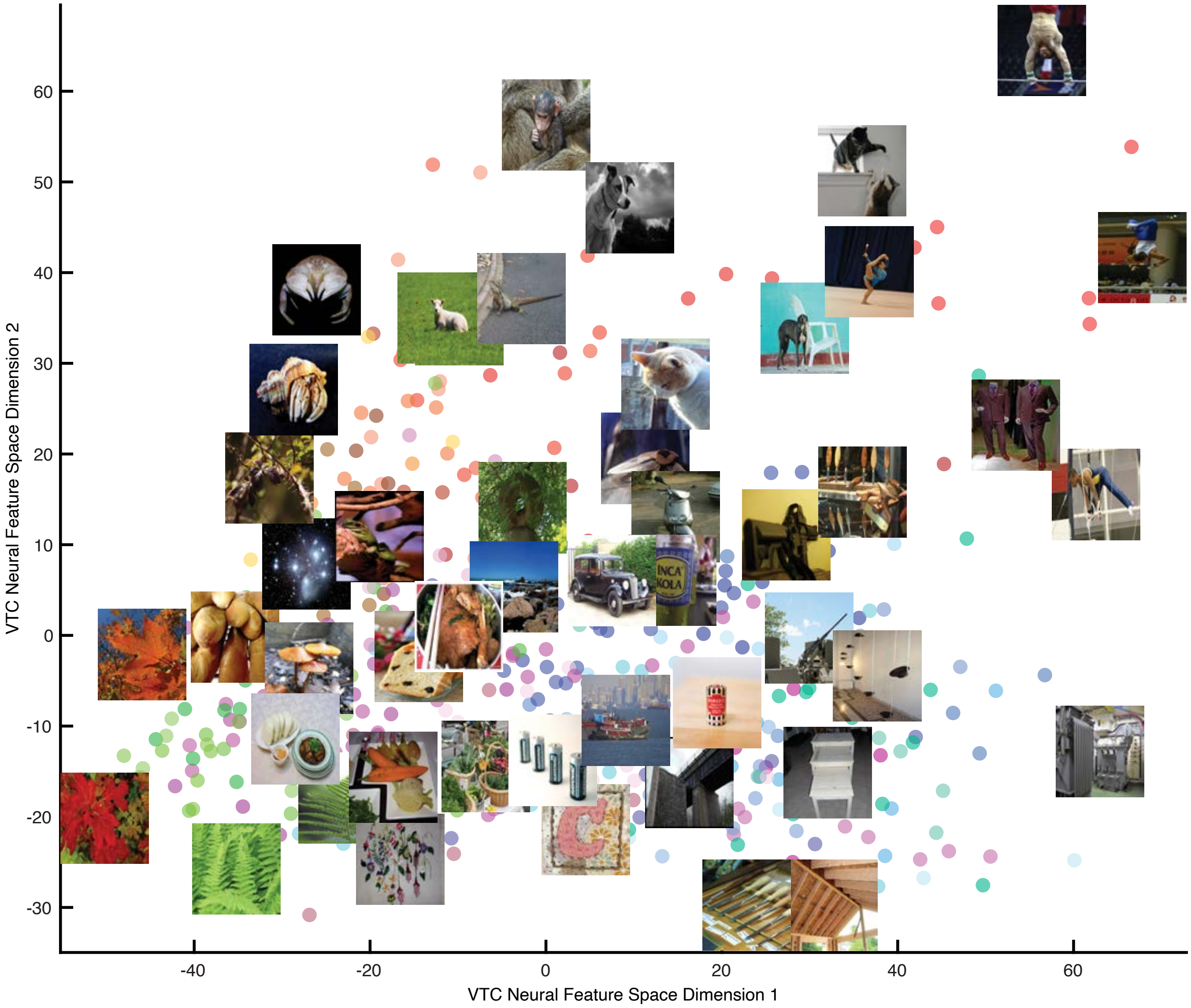

Supplementary Fig. 6. VTC neural feature space with example images. The feature space is the same as that shown in **Fig. 4a**. Colors indicate the broader super categories (e.g., animals, equipment, etc.). Variations within each broader color category represent the nested fine-grained categories. Images were obtained from Deng J, Dong W, Socher R, Li L-J, Li K, Fei-Fei L (2009). IEEE Conference on Computer Vision and Pattern Recognition 2009: 248–55. IEEE. Source data are provided as a Source Data file.

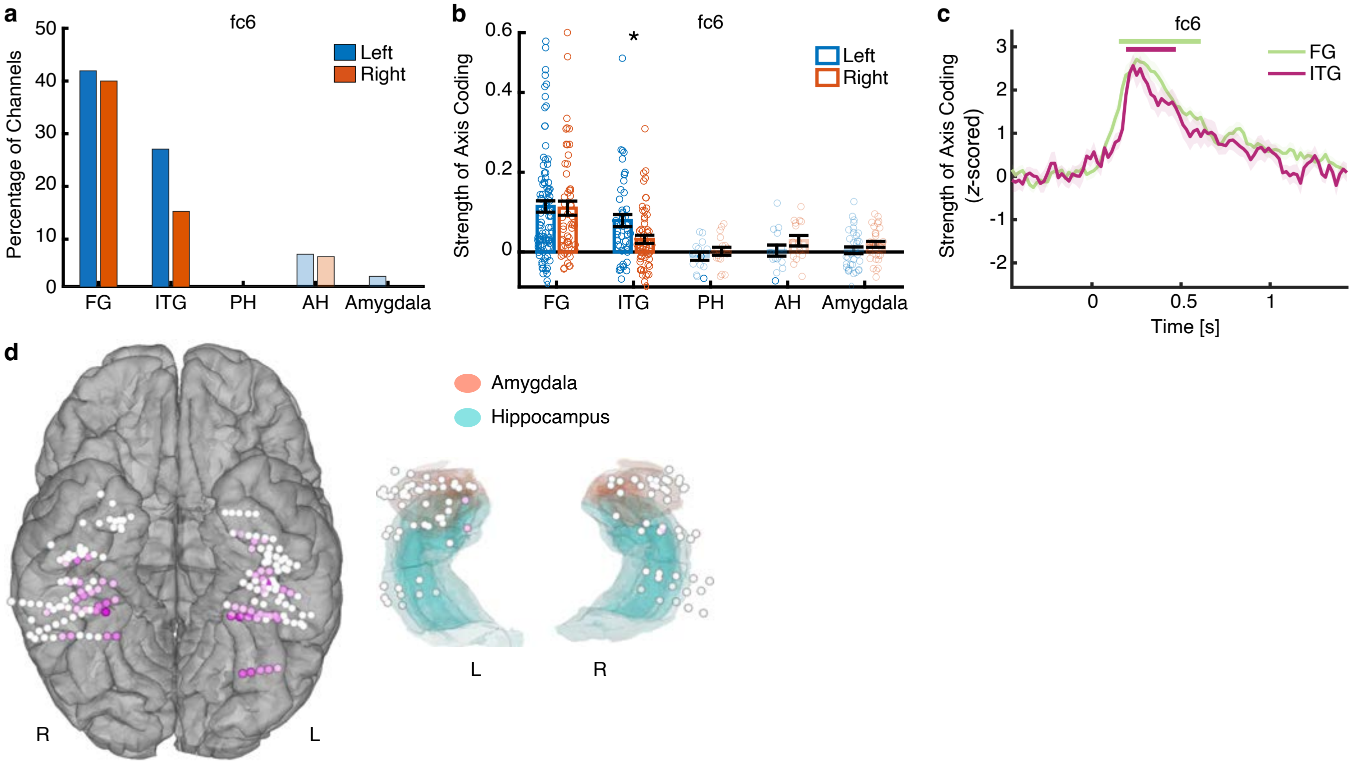

Supplementary Fig. 7. Validation of axis coding using the Microsoft COCO dataset. **(a)** Percentage of channels exhibiting axis coding. **(b)** Strength of axis coding (Pearson's *r*) averaged across all visually responsive channels in each ROI. **(c)** Temporal profile of axis coding among the significant ROIs from the right hemisphere. **(d)** Distribution of axis-coding channels in the VTC and MTL. Legend conventions as in **Fig. 3**. Source data are provided as a Source Data file.

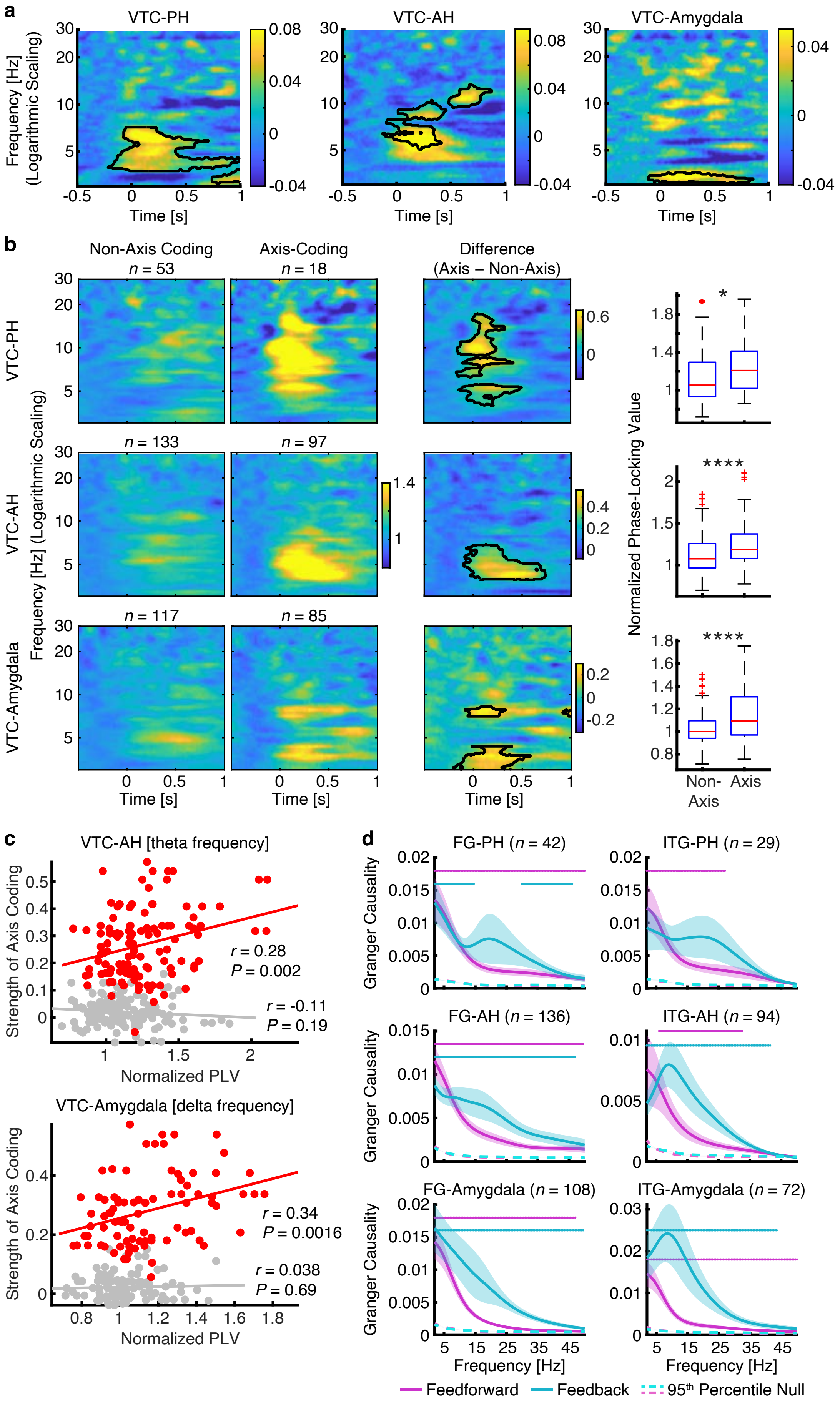

a
VTC-PH
VTC-AH
VTC-Amygdala
Frequency [Hz] (Logarithmic Scaling)
Time [s]
b
Non-Axis Coding
Axis-Coding
Difference (Axis – Non-Axis)
VTC-PH
VTC-AH
VTC-Amygdala
n = 53
n = 18
n = 133
n = 97
n = 117
n = 85
Normalized Phase-Locking Value
Non-Axis
Axis
c
VTC-AH [theta frequency]
Strength of Axis Coding
Normalized PLV
r = 0.28
P = 0.002
r = -0.11
P = 0.19
VTC-Amygdala [delta frequency]
r = 0.34
P = 0.0016
r = 0.038
P = 0.69
d
FG-PH (n = 42)
ITG-PH (n = 29)
FG-AH (n = 136)
ITG-AH (n = 94)
FG-Amygdala (n = 108)
ITG-Amygdala (n = 72)
Granger Causality
Frequency [Hz]
Feedforward
Feedback
95th Percentile Null

Supplementary Fig. 8. Functional connectivity between the ventral temporal cortex (VTC) and medial temporal lobe (MTL). **(a)** Time-resolved phase-locking value (PLV) for representative VTC axis-coding and MTL category-coding channel pairs. The PLV was *z*-scored relative to the pre-trial baseline. The lower row shows only the significant time and frequency points, compared to the null distribution (see **Methods**). **(b, c)** PLV analysis using channel pairs from the ipsilateral side. **(d)** Bidirectional Granger causality (GC) between the VTC and MTL using channel pairs from the ipsilateral side. Legend conventions as in **Fig. 6**. Source data are provided as a Source Data file.

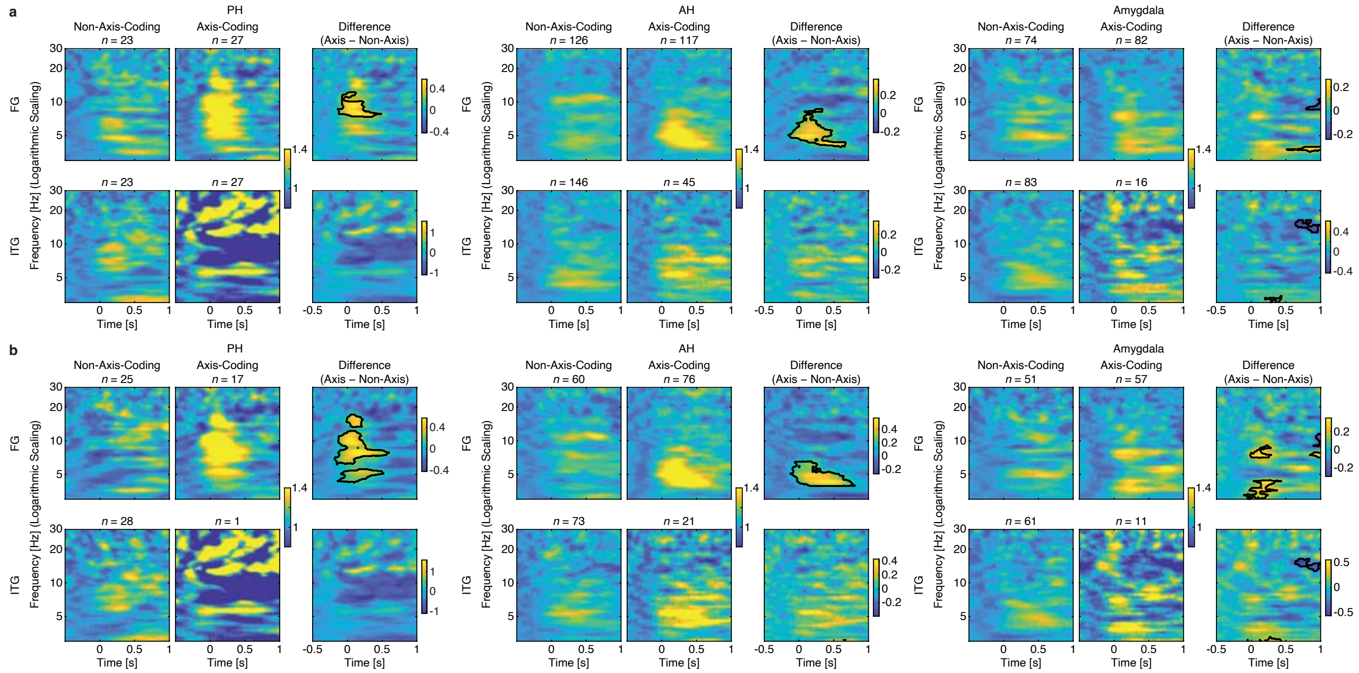

Supplementary Fig. 9. Phase-locking across VTC-MTL subregions. **(a)** Channel pairs from both hemispheres. **(b)** Channel pairs from the ipsilateral hemisphere. Legend conventions as in **Fig. 6b**. Source data are provided as a Source Data file.

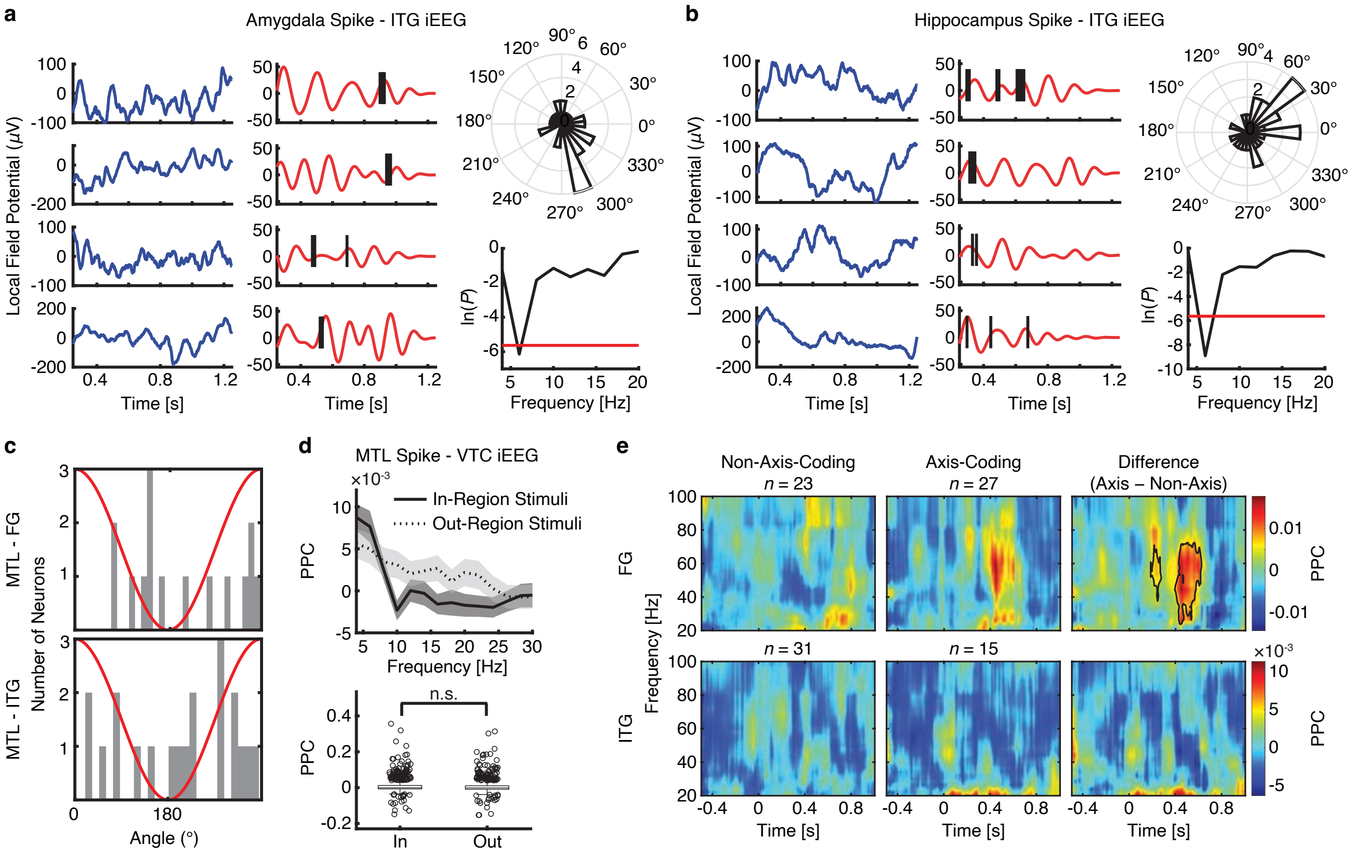

Supplementary Fig. 10. Additional results for spike–iEEG phase-locking. **(a-d)** Spike–iEEG phase-locking in the theta frequency range (4–8 Hz). Legend conventions as in **Fig. 7**. Of the MTL region-coding neurons, 15/35 were phase-locked to the FG and 20/35 to the ITG. In the full population, 102/189 were phase-locked to the FG and 114/189 to the ITG. In contrast to gamma phase-locking, theta phase-locking showed more evenly distributed phases. The pairwise phase coherence (PPC) of the MTL spike–VTC iEEG pairs was not significantly greater for in-region stimuli compared to out-region stimuli in the theta range (two-tailed paired t-test across spike–iEEG pairs: $t(911) = 1.73$, $P = 0.083$, $d = 0.06$, 95% CI = [−0.0004, 0.0062]). n.s.: not significant. **(e)** Time-resolved PPC in the gamma frequency range for axis-coding versus non-axis-coding channels, analyzed separately for the FG (top) and ITG (bottom). Source data are provided as a Source Data file.

Supplementary References

1 Tsao, D. Y., Freiwald, W. A., Knutsen, T. A., Mandeville, J. B. & Tootell, R. B. Faces and objects in macaque cerebral cortex. *Nat Neurosci* **6**, 989-995 (2003). https://doi.org/10.1038/nn1111

2 Yamins, D. L. K. *et al.* Performance-optimized hierarchical models predict neural responses in higher visual cortex. *Proceedings of the National Academy of Sciences* **111**, 8619 (2014). https://doi.org/10.1073/pnas.1403112111

3 Ponce, C. R. *et al.* Evolving Images for Visual Neurons Using a Deep Generative Network Reveals Coding Principles and Neuronal Preferences. *Cell* **177**, 999-1009.e1010 (2019). https://doi.org/10.1016/j.cell.2019.04.005

4 Jonas, J. *et al.* A face-selective ventral occipito-temporal map of the human brain with intracerebral potentials. *Proceedings of the National Academy of Sciences of the United States of America* **113**, E4088-E4097 (2016). https://doi.org/10.1073/pnas.1522033113

5 Vlcek, K. *et al.* Mapping the Scene and Object Processing Networks by Intracranial EEG. *Frontiers in human neuroscience* **14**, 561399 (2020). https://doi.org/10.3389/fnhum.2020.561399

6 Singer, J. J. D., Cichy, R. M. & Hebart, M. N. The Spatiotemporal Neural Dynamics of Object Recognition for Natural Images and Line Drawings. *The Journal of neuroscience : the official journal of the Society for Neuroscience* **43**, 484-500 (2023). https://doi.org/10.1523/JNEUROSCI.1546-22.2022

7 Mel, B. W. SEEMORE: Combining Color, Shape, and Texture Histogramming in a Neurally Inspired Approach to Visual Object Recognition. *Neural Computation* **9**, 777-804 (1997). https://doi.org/10.1162/neco.1997.9.4.777

8 Palmeri, T. J. & Gauthier, I. Visual object understanding. *Nature Reviews Neuroscience* **5**, 291-303 (2004). https://doi.org/10.1038/nrn1364

9 Cichy, R. M., Pantazis, D. & Oliva, A. Resolving human object recognition in space and time. *Nat Neurosci* **17**, 455-462 (2014). https://doi.org/10.1038/nn.3635 http://www.nature.com/neuro/journal/v17/n3/abs/nn.3635.html#supplementary-information

10 Bao, P., She, L., McGill, M. & Tsao, D. Y. A map of object space in primate inferotemporal cortex. *Nature* **583**, 103-108 (2020). https://doi.org/10.1038/s41586-020-2350-5

11 Cohen, U., Chung, S., Lee, D. D. & Sompolinsky, H. Separability and geometry of object manifolds in deep neural networks. *Nature Communications* **11**, 746 (2020). https://doi.org/10.1038/s41467-020-14578-5

12 Hebart, M. N., Zheng, C. Y., Pereira, F. & Baker, C. I. Revealing the multidimensional mental representations of natural objects underlying human similarity judgements. *Nature Human Behaviour* **4**, 1173-1185 (2020). https://doi.org/10.1038/s41562-020-00951-3

13 VanRullen, R. & Reddy, L. Reconstructing faces from fMRI patterns using deep generative neural networks. *Communications Biology* **2**, 193 (2019). https://doi.org/10.1038/s42003-019-0438-y

14 Grossman, S. *et al.* Convergent evolution of face spaces across human face-selective neuronal groups and deep convolutional networks. *Nature Communications* **10**, 4934 (2019). https://doi.org/10.1038/s41467-019-12623-6

15 Bashivan, P., Kar, K. & DiCarlo, J. J. Neural population control via deep image synthesis. *Science* **364**, eaav9436 (2019). https://doi.org/10.1126/science.aav9436

16 Wong, C. & Gallate, J. The function of the anterior temporal lobe: A review of the empirical evidence. *Brain Research* **1449**, 94-116 (2012). https://doi.org/https://doi.org/10.1016/j.brainres.2012.02.017
17 Peelen, M. V. & Caramazza, A. Conceptual Object Representations in Human Anterior Temporal Cortex. *The Journal of Neuroscience* **32**, 15728 (2012). https://doi.org/10.1523/JNEUROSCI.1953-12.2012
18 Bonner, M. F. & Price, A. R. Where Is the Anterior Temporal Lobe and What Does It Do? *The Journal of Neuroscience* **33**, 4213 (2013).
19 Devlin, J. T. *et al.* Susceptibility-induced loss of signal: comparing PET and fMRI on a semantic task. *NeuroImage* **11**, 589-600 (2000). https://doi.org/10.1006/nimg.2000.0595
20 Rossion, B., Jacques, C. & Jonas, J. The anterior fusiform gyrus: The ghost in the cortical face machine. *Neuroscience and biobehavioral reviews* **158**, 105535 (2024). https://doi.org/10.1016/j.neubiorev.2024.105535
21 Kanwisher, N., McDermott, J. & Chun, M. M. The fusiform face area: a module in human extrastriate cortex specialized for face perception. *The Journal of neuroscience : the official journal of the Society for Neuroscience* **17**, 4302-4311 (1997).
22 Pitcher, D., Dilks, D. D., Saxe, R. R., Triantafyllou, C. & Kanwisher, N. Differential selectivity for dynamic versus static information in face-selective cortical regions. *NeuroImage* **56**, 2356-2363 (2011). https://doi.org/10.1016/j.neuroimage.2011.03.067
23 Kreiman, G., Koch, C. & Fried, I. Category-specific visual responses of single neurons in the human medial temporal lobe. *Nat Neurosci* **3**, 946-953 (2000).
24 Quian Quiroga, R., Reddy, L., Kreiman, G., Koch, C. & Fried, I. Invariant visual representation by single neurons in the human brain. *Nature* **435**, 1102-1107 (2005). https://doi.org/http://www.nature.com/nature/journal/v435/n7045/suppinfo/nature03687_S1.html
25 Rutishauser, U. *et al.* Representation of retrieval confidence by single neurons in the human medial temporal lobe. *Nat Neurosci* **18**, 1041-1050 (2015).
26 Rey, H. G. *et al.* Encoding of long-term associations through neural unitization in the human medial temporal lobe. *Nature Communications* **9**, 4372 (2018). https://doi.org/10.1038/s41467-018-06870-2
27 Wang, S., Mamelak, A. N., Adolphs, R. & Rutishauser, U. Encoding of Target Detection during Visual Search by Single Neurons in the Human Brain. *Current Biology* **28**, 2058-2069.e2054 (2018). https://doi.org/https://doi.org/10.1016/j.cub.2018.04.092
28 Reber, T. P. *et al.* Representation of abstract semantic knowledge in populations of human single neurons in the medial temporal lobe. *PLOS Biology* **17**, e3000290 (2019). https://doi.org/10.1371/journal.pbio.3000290
29 Cao, R. *et al.* A neuronal code for object representation and memory in the human amygdala and hippocampus. *Nature Communications* **16**, 1510 (2025). https://doi.org/10.1038/s41467-025-56793-y
30 Cao, R. *et al.* Feature-based encoding of face identity by single neurons in the human amygdala and hippocampus. *Nature Human Behaviour* **9**, 1959-1974 (2025). https://doi.org/10.1038/s41562-025-02218-1
31 Cao, R. *et al.* A neural computational framework for face processing in the human temporal lobe. *Current Biology* **35**, 1765-1778.e1766 (2025). https://doi.org/https://doi.org/10.1016/j.cub.2025.02.063

32 Chang, L. & Tsao, D. Y. The Code for Facial Identity in the Primate Brain. *Cell* **169**, 1013-1028.e1014 (2017). https://doi.org/10.1016/j.cell.2017.05.011

33 Cao, R., Lin, C., Brandmeir, N. J. & Wang, S. A human single-neuron dataset for face perception. *Scientific Data* **9**, 365 (2022). https://doi.org/10.1038/s41597-022-01482-4

34 Cao, R. *et al.* Neural mechanisms of face familiarity and learning in the human amygdala and hippocampus. *Cell Reports* **43**, 113520 (2024). https://doi.org/10.1016/j.celrep.2023.113520

35 Cao, R., Brunner, P., Brandmeir, N. J., Willie, J. T. & Wang, S. A human single-neuron dataset for object recognition. *Scientific Data* **12**, 79 (2025). https://doi.org/10.1038/s41597-024-04265-1
